# Purpura Fulminans Mimicking NSTI: A Limb-saving case of *Haemophilus influenzae* Infection

**DOI:** 10.1016/j.idcr.2026.e02633

**Published:** 2026-06-03

**Authors:** Ryo Watanabe, Akira Kono, Chihiro Narita, Akihiro Miyake, Naoki Tosaka

**Affiliations:** Department of Emergency Medicine, Shizuoka General Hospital, 4-27-1 Kitaando, Aoi Ward, Shizuoka city, Shizuoka 420-0881, Japan

**Keywords:** Purpura fulminans, *Haemophilus influenzae*, Septic cardiomyopathy, VA-ECMO, Limb salvage

## Abstract

**Background:**

Purpura fulminans is a life-threatening thrombotic disorder characterized by disseminated intravascular coagulation and hemorrhagic skin necrosis. Severe cases may be complicated by septic cardiomyopathy requiring veno-arterial extracorporeal membrane oxygenation (VA-ECMO) for circulatory support. The clinical presentation of purpura fulminans closely resembles that of necrotizing soft tissue infections (NSTI), which makes differentiation challenging. Accurate differentiation is crucial for limb preservation strategies.

**Case Presentation:**

A 52-year-old man with a history of hypertension presented with hypothermia, widespread purpura, and severe bilateral leg pain. Suspecting NSTI, exploratory incisions were performed, revealing asymmetric tissue findings. Despite maximal vasopressor support, he remained hemodynamically unstable and required VA-ECMO. During ECMO management, the patient developed compartment syndrome requiring fasciotomy. Blood and wound cultures grew *Haemophilus influenzae*. With multidisciplinary management, including prone positioning and fluid removal with continuous renal replacement therapy (CRRT), ECMO was successfully weaned on day 3. Although persistent infection and rhabdomyolysis-induced acute kidney injury (AKI) necessitated amputation of the left leg, the right leg was salvaged with skin grafting. The patient was eventually weaned from dialysis and discharged ambulatory with a prosthetic limb after rehabilitation.

**Conclusions:**

This case highlights the diagnostic challenge of distinguishing purpura fulminans from NSTI and emphasizes the importance of early ECMO weaning to improve patient outcomes. A multidisciplinary strategy integrating circulatory stabilization, fasciotomy, and careful limb preservation may optimize survival and functional recovery in patients with severe purpura fulminans. Differentiating between purpura fulminans and NSTI is crucial for preventing unnecessary amputations and preserving limb function.

## Highlights

• A rare case of purpura fulminans caused by *Haemophilus influenzae* is presented.
• The patient developed septic cardiomyopathy and required VA-ECMO support.
• One leg was salvaged through multidisciplinary management and delayed amputation.

## Introduction

Purpura fulminans is a rapidly progressive and life-threatening thrombotic disorder characterized by widespread hemorrhagic necrosis of the skin and disseminated intravascular coagulation (DIC) [1], [2]. Its clinical presentation often overlaps with NSTI, making early differentiation challenging. The incidence of NSTI has increased in recent years, further complicating the timely and accurate diagnosis of this condition in critically ill patients.

A timely diagnosis is crucial for limb preservation because aggressive surgical intervention is necessary for NSTI, but it can be harmful for purpura fulminans. In this case, the patient developed septic shock with signs concerning for NSTI. However, careful evaluation, including serial exploratory incisions and pathogen identification, allowed for appropriate conservative management. In this case, despite severe ischemic changes in both lower limbs, a multidisciplinary approach including circulatory support, fasciotomy, and strategic fluid management enabled successful stabilization and preservation of one leg.

This case emphasizes the importance of differentiating purpura fulminans from NSTI to prevent unnecessary amputations. It also highlights the significance of early, tailored interventions in improving survival rates and preserving limbs.

## Case

**Fig. 1:**
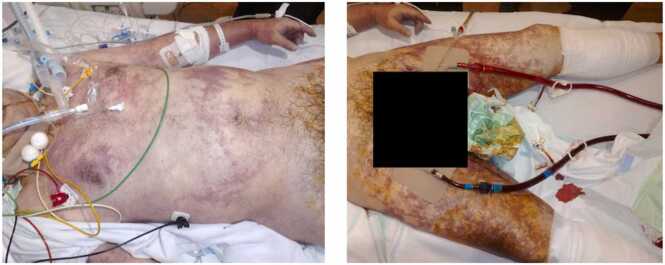
Clinical photograph taken on admission, showing widespread purpura over the entire body.

A 52-year-old man was transported to the emergency department with chief complaints of hypothermia and bilateral leg paresthesia. He had reported cold-like symptoms, including sore throat and fever, since the previous day. The next morning, his family found him unable to move. His only past medical history was hypertension. The patient was not immunocompromised. He did not receive the *Haemophilus influenzae* type b (Hib) vaccine as a child. Upon arrival, the patient had purpura primarily on his lower extremities, but also over his entire body. He was in shock with hypothermia. Warming, fluid resuscitation, and catecholamine administration were initiated. Empirical intravenous antimicrobial therapy with meropenem (2 g every 8 h), levofloxacin (750 mg every 48 h), vancomycin (a loading dose of 2400 mg followed by 500 mg every 24 h), and minocycline (100 mg every 12 h) was initiated immediately after blood cultures were obtained (Fig 1).

Despite maximal vasopressor support, he remained hemodynamically unstable, which was thought to be due to septic cardiomyopathy. High-flow nasal cannula (HFNC) oxygen therapy was started due to poor oxygenation. The patient complained of severe pain in both lower extremities, most intensely in the right heel. We performed exploratory incisions, suspecting NSTI. The findings revealed poor bleeding from the tissues of the right leg, tissue fragility, and discoloration of the fascia. In contrast, the fascia of the left leg appeared well perfused with active bleeding, indicating a difference between the two sides. Bacterial cultures of exudates from both legs were submitted. Gram stain revealed the presence of gram-negative rods (GNR), and the rapid test for group A streptococcus (GAS) was negative. Although amputation was considered for NSTI, the negative GAS test result and the presence of purpura on both legs led to a decision against immediate amputation. A CT scan revealed splenic atrophy, indicating hyposplenism and predisposing the patient to infection.

Six hours later, despite high-dose vasopressor support (norepinephrine at 0.6 μg/kg/min, vasopressin at 2.4 U/h, and epinephrine at 0.2 μg/kg/min) and an FiO₂ of 0.6, the patient experienced rapid cardiopulmonary deterioration. Transthoracic echocardiography revealed a reduced left ventricular ejection fraction (EF) of 35%, which necessitated intubation and the initiation of VA-ECMO. The VA-ECMO configuration consisted of an 18 French (Fr) arterial return cannula placed in the left femoral artery and a 22 Fr venous drainage cannula inserted via the right femoral vein.

On the second day, cardiac function showed signs of improvement. However, fluid resuscitation had resulted in severe pulmonary edema and persistent hypoxemia. Because further improvement in cardiac function was anticipated, weaning from ECMO was postponed until the following day. To prepare for ECMO weaning, we used CRRT and diuretics to achieve a negative fluid balance and implemented prone positioning to improve oxygenation.

A second-look surgery was performed on both lower legs, which had undergone exploratory incisions the previous day. Although no progression of necrosis was observed, the left lower leg became increasingly swollen. A fasciotomy was performed after elevated intracompartmental pressure was confirmed by compartment pressure measurement. Blood cultures and wound exudate from the exploratory incisions grew *Haemophilus influenzae*. Subsequent susceptibility testing revealed that the isolate was β-lactamase–negative and susceptible to ceftriaxone and levofloxacin. Based on these results, antimicrobial therapy was de-escalated to intravenous ceftriaxone (2 g every 24 h) and levofloxacin. Due to the critical condition of the patient, dual antimicrobial coverage was maintained throughout the acute phase.

**Fig. 2:**
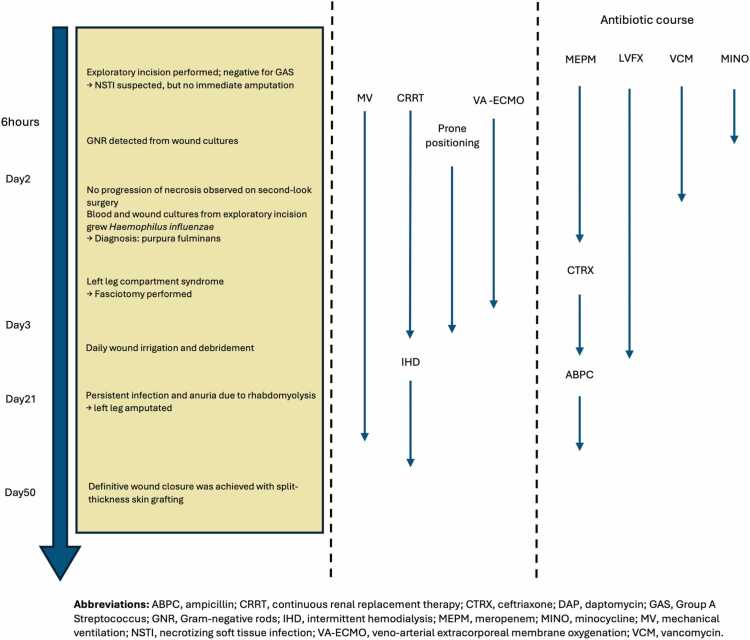
Clinical timeline. A chronological overview of the patient's course.

**Fig. 3:**
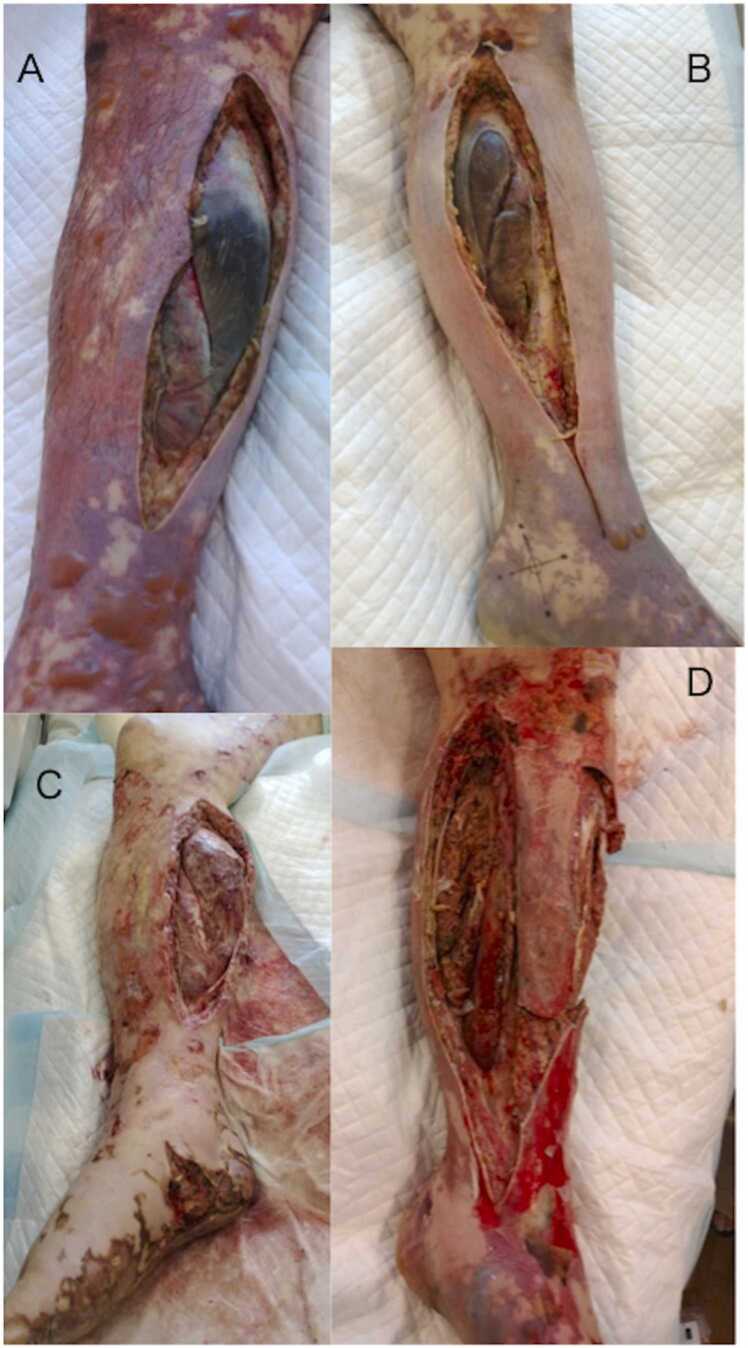
Clinical photographs of the patient taken on hospital day 6 (A, B) and day 13 (C, D). The left lower extremity showed poorer perfusion compared to the right, with progressive necrosis over time, ultimately requiring amputation.

On day 3, the VA-ECMO was successfully weaned. At that time, the patient was receiving epinephrine at a rate of 0.2 μg/kg/min. Subsequent management included daily wound irrigation and debridement of necrotic skin and muscle in both lower extremities. However, by day 21, controlling the infection in the left lower extremity remained difficult and the patient continued to experience anuria due to rhabdomyolysis-induced AKI. Consequently, the left lower extremity was amputated to manage the infection, rhabdomyolysis, and AKI (Fig. 2, Fig. 3).

**Fig. 4:**
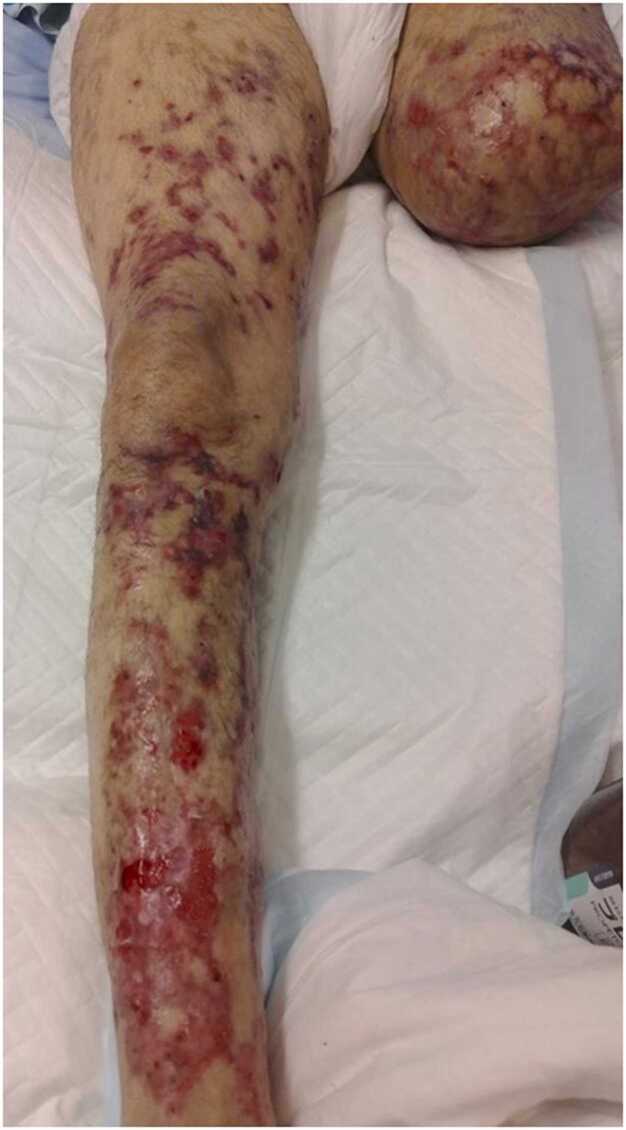
Clinical photograph showing the condition after amputation of the left lower extremity and skin grafting on the right lower extremity.

The amputation site had some necrotic skin, raising concerns about wound dehiscence and infection. However, the postoperative course was uneventful. Wound irrigation and debridement of the remaining right lower extremity continued. Negative pressure wound therapy was also applied to promote granulation. Definitive wound closure was achieved with split-thickness skin grafting on day 50. Amputating the left limb contributed to the patient's successful recovery, enabling him to be weaned from renal replacement therapy and retain function in his right lower extremity. He was subsequently transferred to a rehabilitation facility. 10 months later, he was discharged from the rehabilitation hospital ambulatory with a prosthetic limb (Fig. 4).

## Discussion

This case highlights the challenges in diagnosing and treating purpura fulminans, a rare and potentially fatal thrombotic disorder. A previously healthy adult presented with sudden-onset hypothermia, hypotension, and rapidly progressing purpura on the lower extremities. On admission, the patient was in septic shock and had extensive purpuric lesions on both legs, raising initial concerns for NSTI. Despite aggressive resuscitation efforts, the patient developed refractory shock. Six hours after admission, ECMO was initiated for cardiopulmonary support. By day 2, cardiac function had improved, but severe pulmonary edema persisted, requiring prolonged ventilatory support. Careful fluid management through CRRT and prone positioning enabled early ECMO weaning. Fasciotomy was performed to alleviate compartment syndrome that developed during ECMO support. Amputation decisions were guided by serial assessments of limb perfusion and tissue viability. The left leg showed progressive necrosis and persistent anuria due to infection and rhabdomyolysis, which required amputation on day 21. In contrast, the right leg showed gradual improvement in perfusion, enabling successful limb preservation. Following stabilization, the patient underwent split-thickness skin grafting to cover the wound and was discharged with preserved mobility.

To our knowledge, only a few cases of purpura fulminans in adults due to *Haemophilus influenzae* requiring ECMO support have been reported in the literature (Table 1). Reports of survival to hospital discharge with limb preservation following ECMO support are extremely rare [3], [4], [5], [6]. Our case is unique in demonstrating that aggressive circulatory support, timely surgical interventions, and a limb-preserving strategy can lead to a favorable outcome even in severe purpura fulminans.

**Table 1:** Reported adult cases of purpura fulminans caused by *Haemophilus influenzae*.

Author (year)	Age	Sex	Immune status	VA-ECMO	Amputation	Survival
Gast (2005)	34	Female	Immunocompetent	Yes	Bilateral	Survived
Endo (2014)	41	Male	Immunocompetent (splenic atrophy)	Yes	Bilateral	Died
Bhika Beechar (2020)	53	Male	Immunocompetent	No	None	Survived
Oliveira Miranda (2024)	58	Female	Mildly immunocompromised	No	Bilateral	Survived
Current case	52	Male	Immunocompetent (splenic atrophy)	Yes	Unilateral	Survived

Abbreviations: VA-ECMO, veno-arterial extracorporeal membrane oxygenation.

In recent years, NSTI has become widespread worldwide, including Japan, with a notable increase in invasive group A streptococcal infections [7], [8], [9]. Both NSTI and purpura fulminans present with purpura, especially on the limbs, as well as septic shock. This makes it difficult to differentiate between the two during initial treatment. In NSTI, rapid systemic deterioration is primarily due to widespread bacterial invasion and tissue necrosis. This necessitates early surgical intervention to prevent fatal progression [10], [11]. In contrast, purpura fulminans is characterized by microvascular thrombosis and DIC, resulting in ischemic necrosis. Tissue damage in purpura fulminans occurs more slowly due to the gradual development of microthrombi, which allows more time to assess limb viability before amputation is decided upon [12]. Studies have shown that the median time to amputation of a limb in NSTI is 4.5 days, whereas in purpura fulminans, it is 18 (14–28 IQR) days. This highlights the longer decision-making window in the latter condition. Therefore, early differentiation between these two conditions is crucial, as purpura fulminans may allow for a more cautious approach to limb preservation [11], [12].

In this case, the patient presented with purpura and pain in the lower extremities. There was marked discoloration and tissue fragility in the right leg compared to the left, leading to the initial consideration of NSTI. GNR was cultured from wound exudates on both legs, a finding that has also been reported in purpura fulminans. This further complicated the differential diagnosis with NSTI [13]. However, the patient was ultimately diagnosed with acute infectious purpura fulminans rather than NSTI. This diagnosis was based on the clinical course, serial surgical assessments including second-look operations, the absence of rapidly progressive fascial necrosis requiring extensive debridement, and the gradual demarcation pattern of ischemic skin lesions consistent with microvascular thrombosis. In the differential diagnosis between NSTI and purpura fulminans, especially when the lesions are primarily on the lower limbs, findings from exploratory incisions and differences in pain intensity or tissue discoloration between the legs may be subjective and insufficient for definitive diagnosis. In such cases, immediate amputation based solely on initial findings should be avoided. Waiting for microbiological identification of the causative organism can help prevent unnecessary amputations and lead to safer treatment decisions. It is important to note that a single exploratory incision may not provide sufficient information, and a planned second-look operation is essential to assess necrosis progression and guide appropriate surgical intervention [14].

The management of purpura fulminans, particularly when it leads to systemic deterioration, should prioritize treating the underlying sepsis and coagulopathy. In some cases, ECMO may be required to save the patient. Microvascular thrombosis due to coagulopathy often leads to significant limb ischemia in purpura fulminans. ECMO further increases the risk of ischemia. Additionally, increased vascular permeability and aggressive fluid resuscitation for septic shock often contribute to compartment syndrome in cases of purpura fulminans [15]. Fasciotomy is useful when limb ischemia is a concern [12]. Thus, in cases of purpura fulminans or NSTI, limb preservation remains an important issue even after systemic stabilization. Prolonged ECMO use may result in further limb damage, requiring careful monitoring. Early ECMO weaning, supported by a multidisciplinary approach that includes prone positioning, can play a critical role in limb salvage. The timing of amputation is also important. Amputating too early can lead to unnecessary limb loss, while waiting too long can lead to secondary infection from necrotic tissue and renal damage due to rhabdomyolysis. In this case, decisions regarding limb preservation were made based on muscle contraction, color, laboratory results, and vital signs. After persistent anuria due to rhabdomyolysis, the left lower extremity was amputated on day 21. Following amputation, improvements in infection control and renal function enabled the right leg to be preserved and successfully treated with a skin graft.

Although our patient had no known immunodeficiency, several factors may have contributed to the development of an invasive *Haemophilus influenzae* infection, which resulted in purpura fulminans. First, he had not received the Hib vaccine during childhood because routine Hib immunization had not yet been introduced in Japan at the time of his birth. The lack of prior immunization may have reduced the patient's protective immunity against encapsulated strains of *Haemophilus influenzae*.

Additionally, functional or anatomical asplenia is a recognized risk factor for severe infection caused by encapsulated organisms. Patients without splenic function experience impaired opsonization and clearance of encapsulated bacteria, resulting in an increased risk of invasive infections. A large observational study demonstrated that reduced splenic volume on computed tomography scans was independently associated with increased mortality in patients with sepsis, suggesting that splenic size may reflect host immune competence in critical illness [16]. Furthermore, patients with asplenia are particularly susceptible to severe infections caused by encapsulated pathogens, including *Haemophilus influenzae*, *Streptococcus pneumoniae*, and *Neisseria meningitidis* [17]. Although our patient had no history of splenectomy, imaging revealed splenic atrophy that functionally resembled hyposplenism. This condition may have contributed to the patient's susceptibility to an invasive *Haemophilus influenzae* infection and subsequent purpura fulminans.

## Conclusion

This is a case of a patient who developed purpura fulminans, caused by *Haemophilus influenzae*, which was complicated by septic cardiomyopathy. This required the initiation of VA-ECMO. The recent increase in NSTI cases made differentiating between the two conditions particularly challenging. In this case, the results of a second-look and wound culture results played a critical role in confirming the diagnosis. When VA-ECMO is required for purpura fulminans, early weaning is desirable for limb preservation, and multidisciplinary management is essential to achieve this. Throughout the clinical course, limb preservation should be carefully considered, balancing the risks of early versus delayed amputation.

## CRediT authorship contribution statement

**Ryo Watanabe:** Writing – original draft, Investigation, Conceptualization. **Akira Kono:** Writing – review & editing, Supervision. **Chihiro Narita:** Writing – review & editing, Supervision. **Akihiro Miyake:** Project administration. **Naoki Tosaka:** Project administration.

## Consent

Written informed consent was obtained from the patient for publication of this case report and accompanying images.

## Ethical approval

Ethical approval was waived for this case report in accordance with institutional policy.

## Funding source

This research did not receive any specific grant from funding agencies in the public, commercial, or not-for-profit sectors.

## Declaration of Competing Interest

The authors declare that they have no known competing financial interests or personal relationships that could have appeared to influence the work reported in this paper.
